# Supplementary figures and images for: Progeny counter mechanism in malaria parasites is linked to extracellular resources
Source: PLoS Pathog. 2023 Dec 5;19(12):e1011807. doi: 10.1371/journal.ppat.1011807 (PMC10723702; doi:10.1371/journal.ppat.1011807)

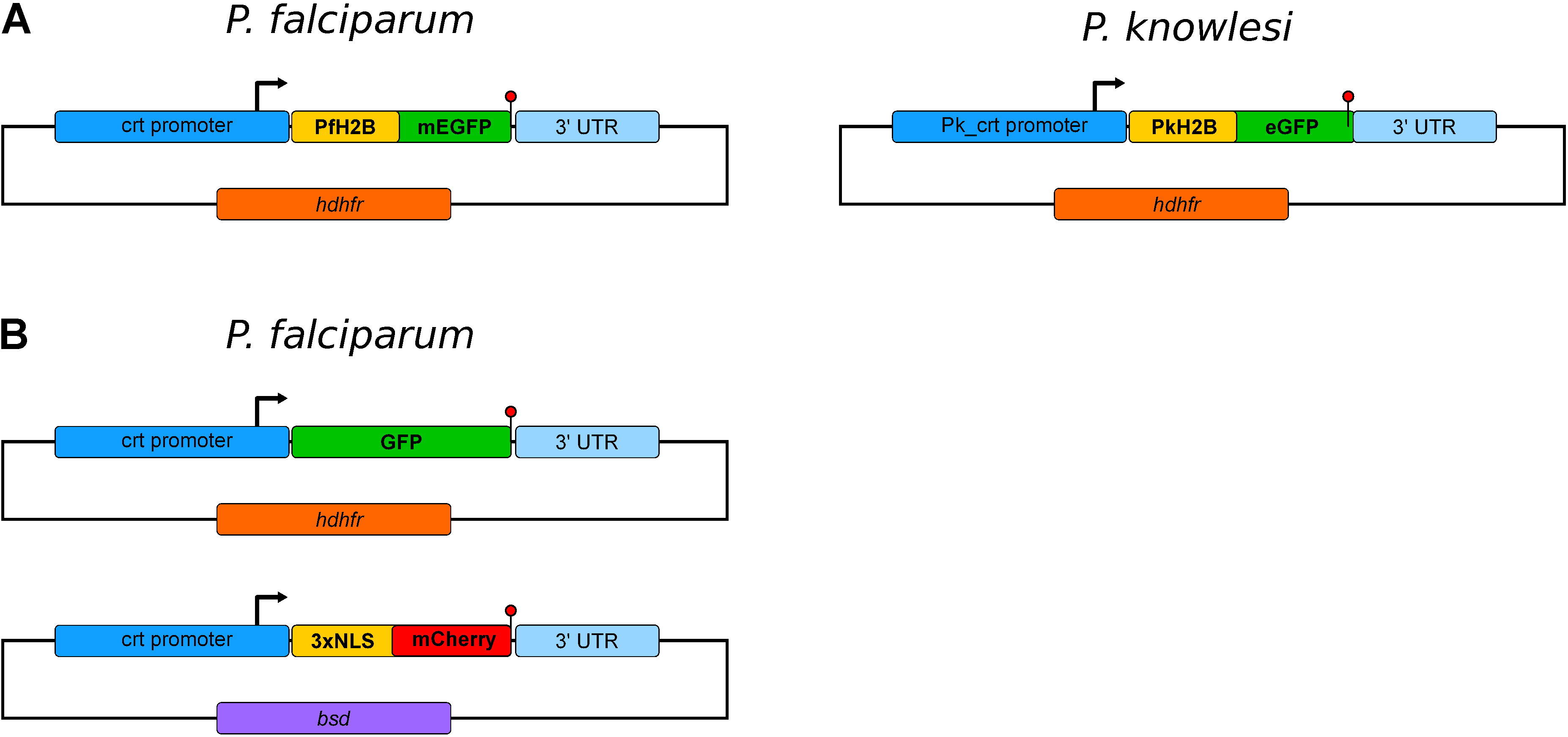

Supplement: S1 Fig — A Two versions of Histone-2B-GFP expressing plasmids used for transfection of P. falciparum and P. knowlesi, respectively. B Two plasmids used for double transfection of P. falciparum for cell and nuclear volume analysis. (TIF) [file ppat.1011807.s001.tif]

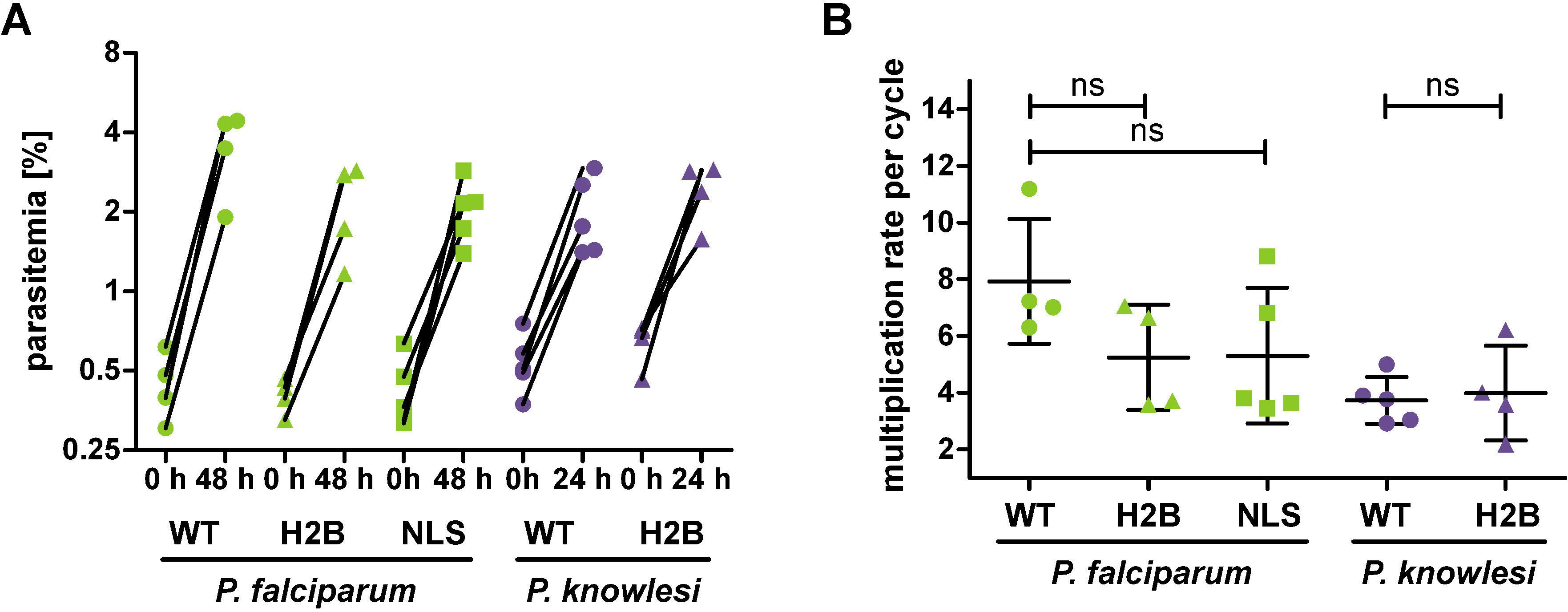

Supplement: S2 Fig — A Parasitemia of wild type and overexpression lines was assessed 48 h or 24 h apart for asynchronous P. falciparum or P. knowlesi cultures, respectively. 4 or 5 replicas were counted per condition. B Datapoints from A converted into multiplication rate per cycle are not different between P. falciparum or P. knowlesi lines. Black bars represent mean and SD. Statistical analysis: t-test with Welch’s correction. (TIF) [file ppat.1011807.s002.tif]

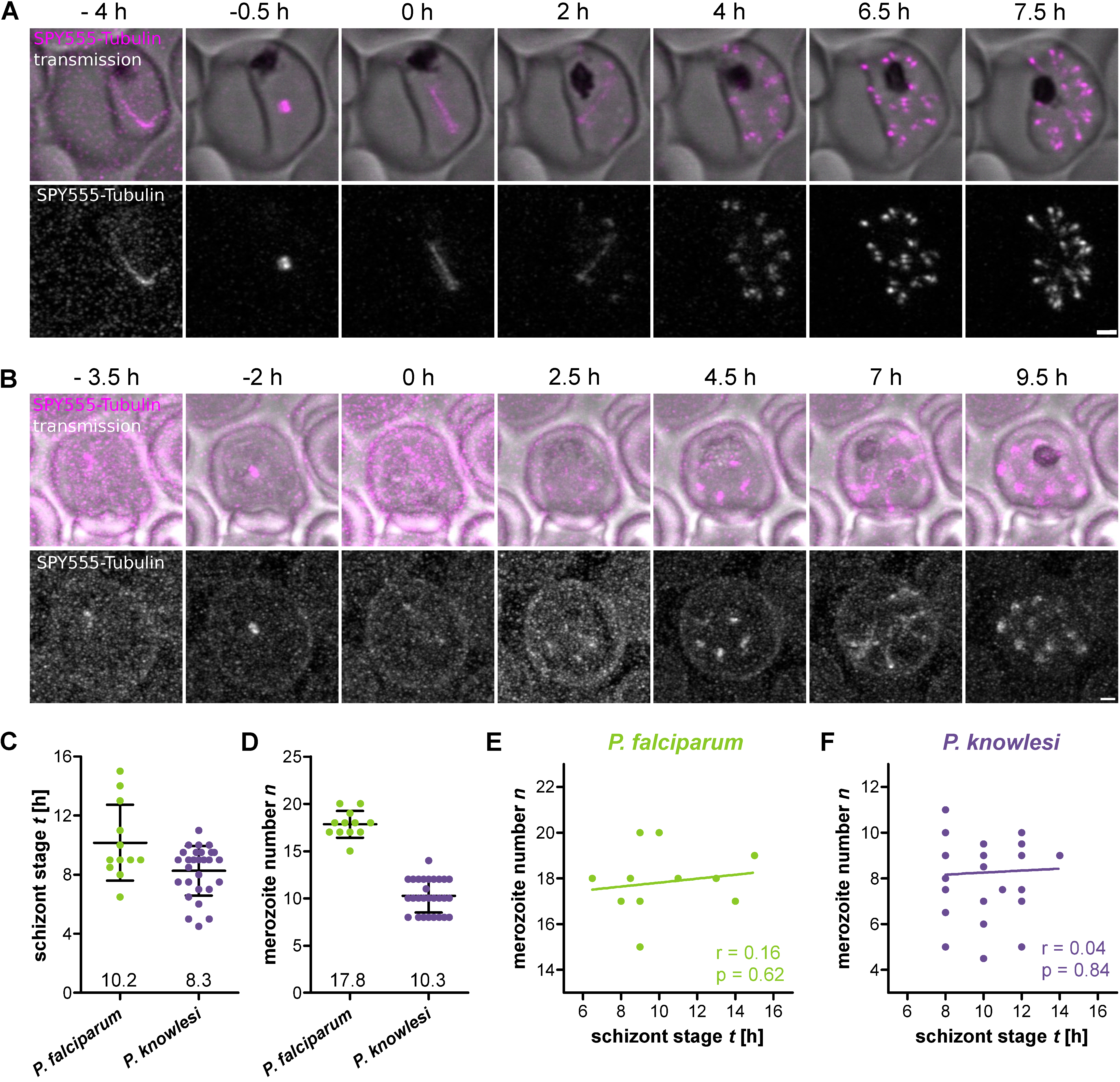

Supplement: S3 Fig — A Airyscan-processed time-lapse images of P. falciparum strain 3D7 stained with SPY555-Tubulin. Timepoint of first spindle elongation was set to 0 h and the timepoint where segmentation started was considered as schizogony end. Shown are maximum intensity projections. Scale bar is 1 μm. B Same as A but for P. knowlesi strain A1-H.1. C Quantification of schizont stage duration in hours and D final merozoite number based on number of subpellicular microtubule structures. Black bars represent mean and SD. E Correlation of merozoite number against duration of schizont stage for P. falciparum. N = 12. Given are Pearson correlation coefficient r and p values. F Same as E but for P. knowlesi. N = 28. (TIF) [file ppat.1011807.s003.tif]

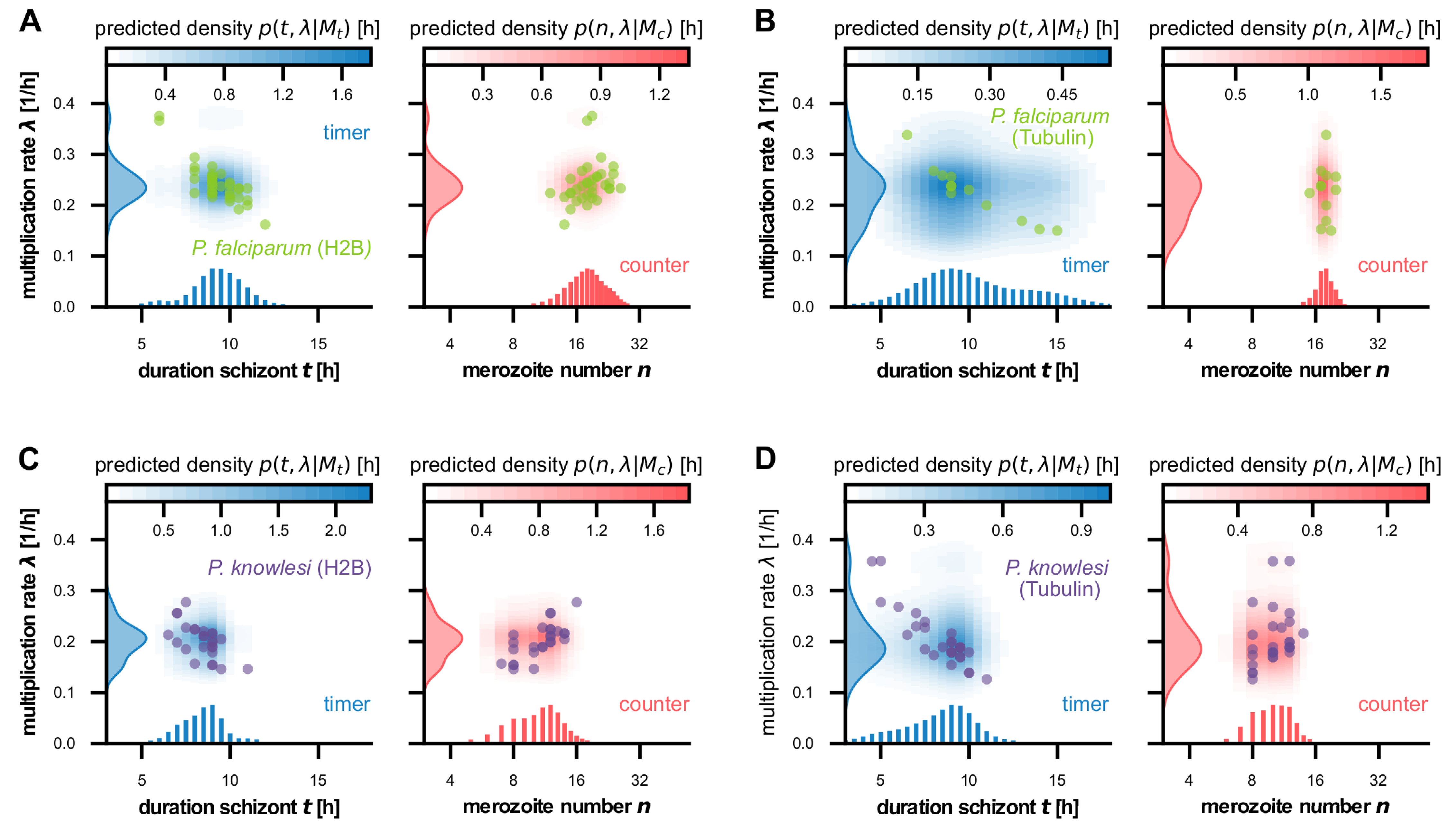

Supplement: S4 Fig — A Growth rate λ vs. duration t (left) and vs. merozoite number n (right) for P. falciparum H2B-GFP expressing schizonts. The timer model density (blue) is constructed from the marginals for λ and t (inset, blue) assuming their independence. The counter model density (red) is constructed similarly from the marginals for λ and n (inset, red). B-D As (A) but for the other strains as indicated. (TIF) [file ppat.1011807.s004.tif]

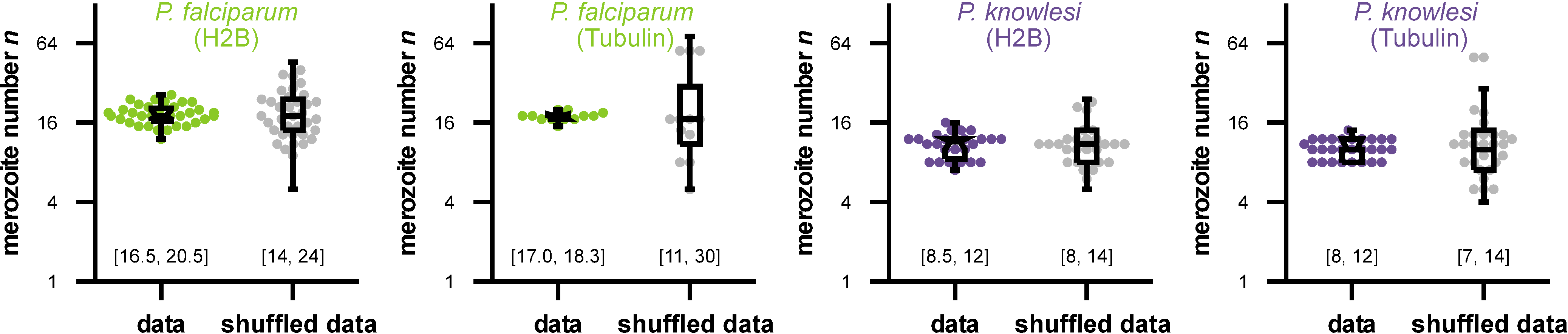

Supplement: S5 Fig — Merozoite number data as in Fig 1E but for all SPY555-Tubulin labelled and H2B-GFP expressing strains, alongside synthetic data generated by randomly reassigning t and λ pairs, followed by recalculating n according to the timer model. Shown are SD and quartiles, and shuffled data points as a swarm plot. Variation of shuffled data is higher as indicated by the interquartile ranges below. (TIF) [file ppat.1011807.s005.tif]

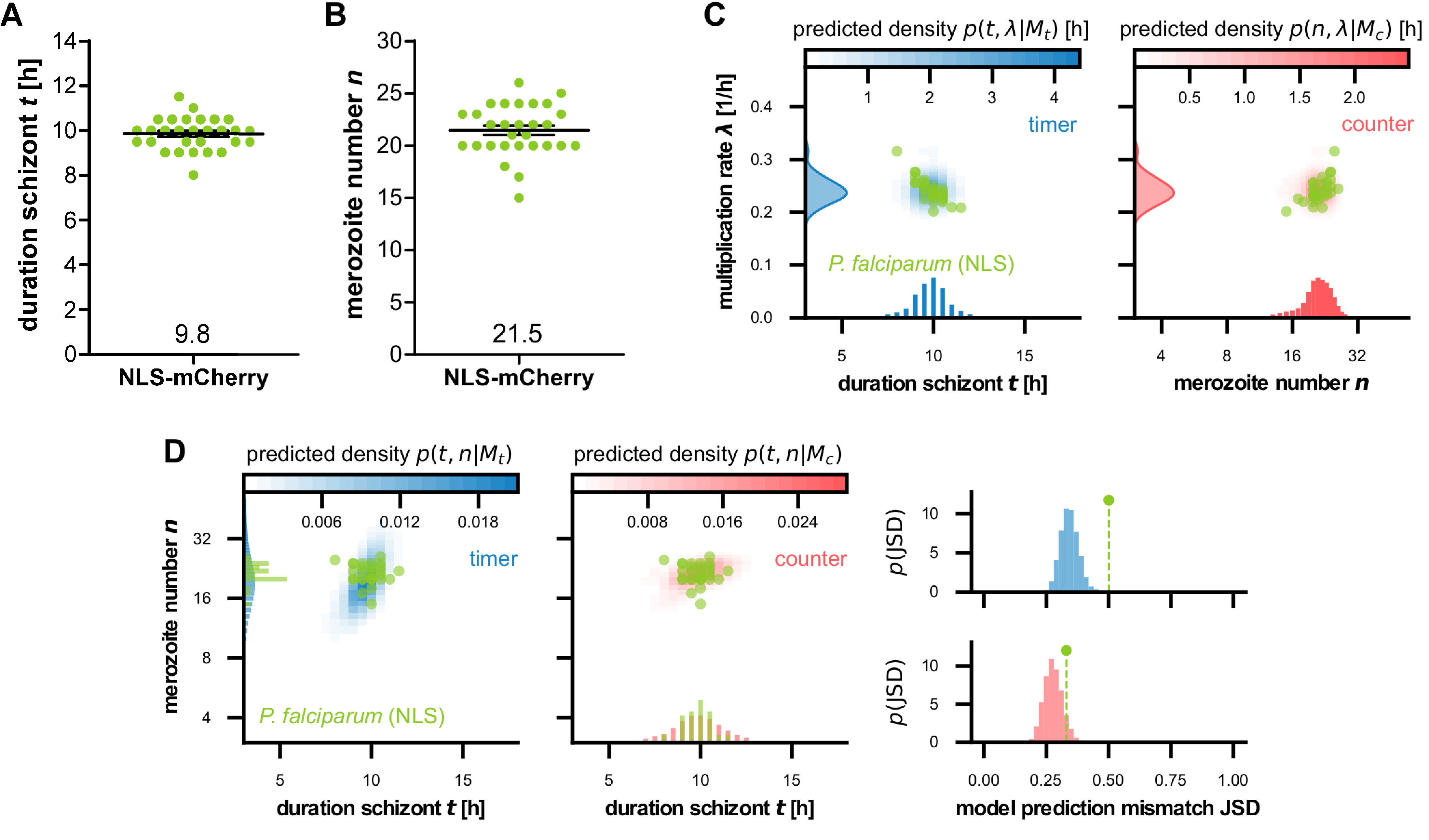

Supplement: S6 Fig — A Quantification of schizont stage duration in hours and B merozoite number of P. falciparum 3D7 episomally expressing a nuclear mCherry signal (NLS-mCherry). Given are means and SEM. C Growth rate λ vs. duration t (left) and vs. merozoite number n (right) for P. falciparum NLS-mCherry and cytoplasmic GFP expressing schizonts. The timer model density (blue) is constructed from the marginals for λ and t (inset, blue) assuming their independence. The counter model density (red) is constructed similarly from the marginals for λ and n (inset, red). D Measured number vs. duration. Timer (blue, left) and counter (red, right) predict different densities in the t−n plane and marginal distribution in n and t, respectively, based on the data. Jensen-Shannon distance (JSD) between data and model density (marker) for timer and counter (top and bottom, respectively). JSD histograms for synthetic data sampled from timer (blue) and counter (red) models show that the data are representative of a counter but not timer sample. (TIF) [file ppat.1011807.s006.tif]

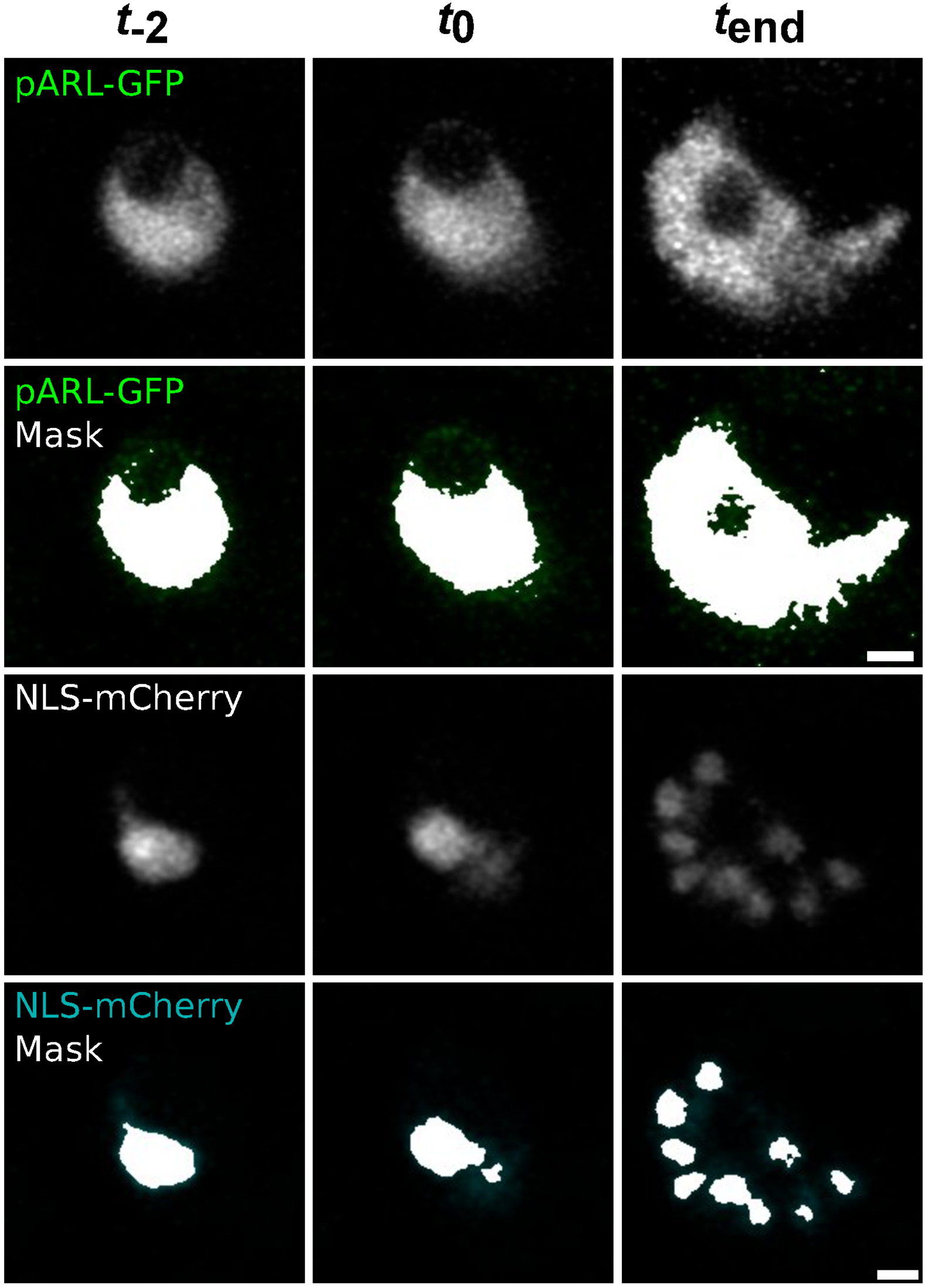

Supplement: S7 Fig — Exemplary single image slices from time points pre-schizogony (t−2), schizogony start (t0) and schizogony end (tend) showing cytoplasmic marker pARL-GFP (green), nuclear marker NLS-mCherry (cyan), and thresholding masks generated for measuring respective areas and volumes. GFP mask was determined by automatic thresholding and mCherry by manual and visual adjustment. Shown is only z slice 11 of a stack of 20 slices to demonstrate segmentation, while analysis was carried out on the entire stack, which then contains all the nuclei. Contrast was adjusted for better visualization. Scale bar is 1 μm. (TIF) [file ppat.1011807.s007.tif]

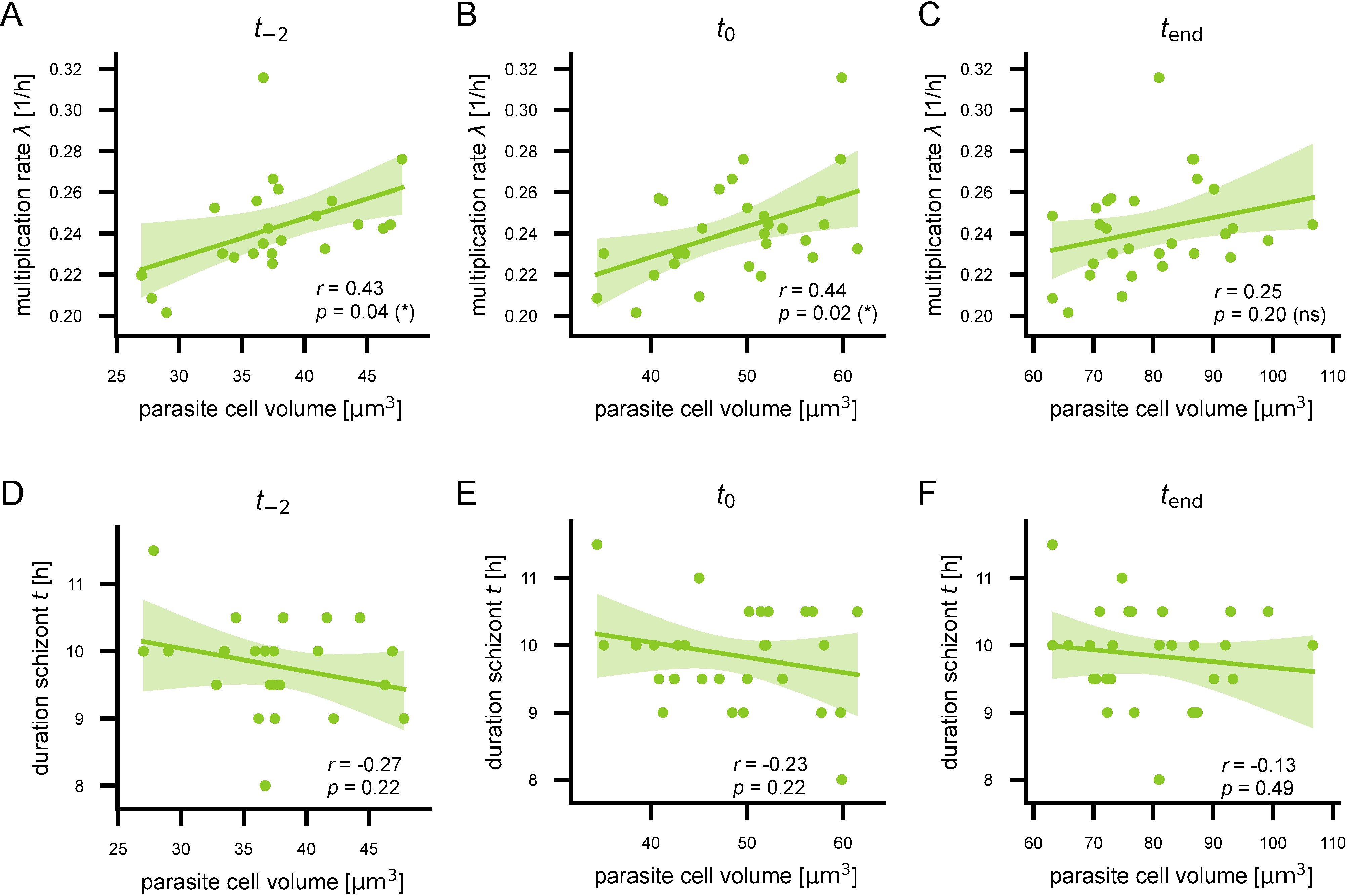

Supplement: S8 Fig — Show are regression curves of cellular parameters t and λ measured in P. falciparum 3D7 episomally expressing a nuclear mCherry signal and cytoplasmic GFP plotted against parasite cell volume measured at replication start, schizont stage onset, and schizont stage end. N = 23 for t−2 and N = 29 for all others from three independent replicates. Given are Pearson correlation coefficient r and p values. Values are bootstrapped to 95% confidence interval. (TIF) [file ppat.1011807.s008.tif]

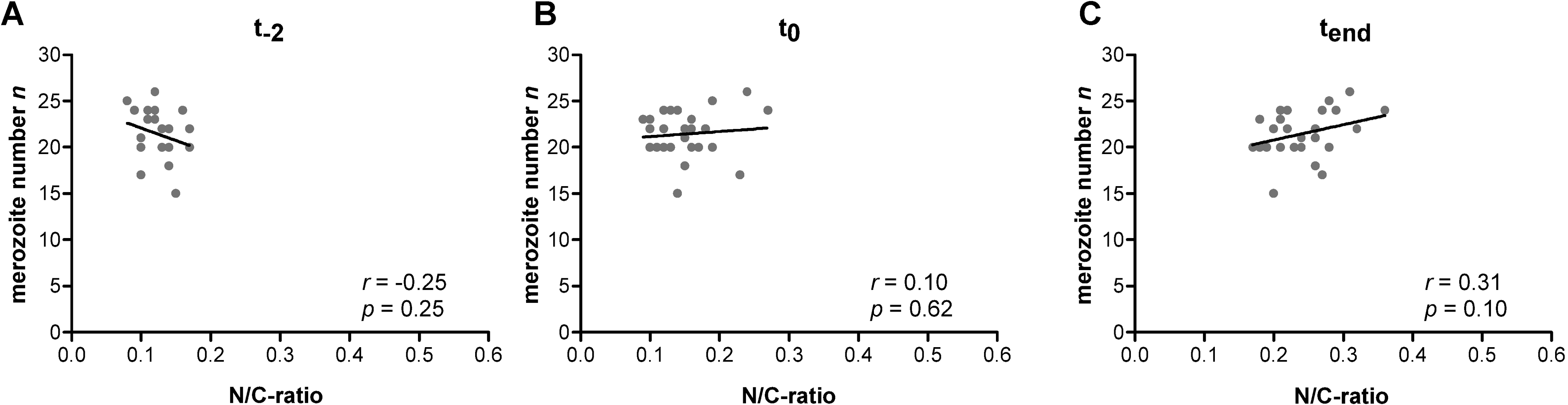

Supplement: S9 Fig — Correlation of merozoite number against N/C ratio all for A pre-schizogony (t−2), B schizogony start (t0) and C schizogony end (tend). Given are Pearson correlation coefficient r and p values. N = 23 for t−2 and N = 29 for all others. (TIF) [file ppat.1011807.s009.tif]

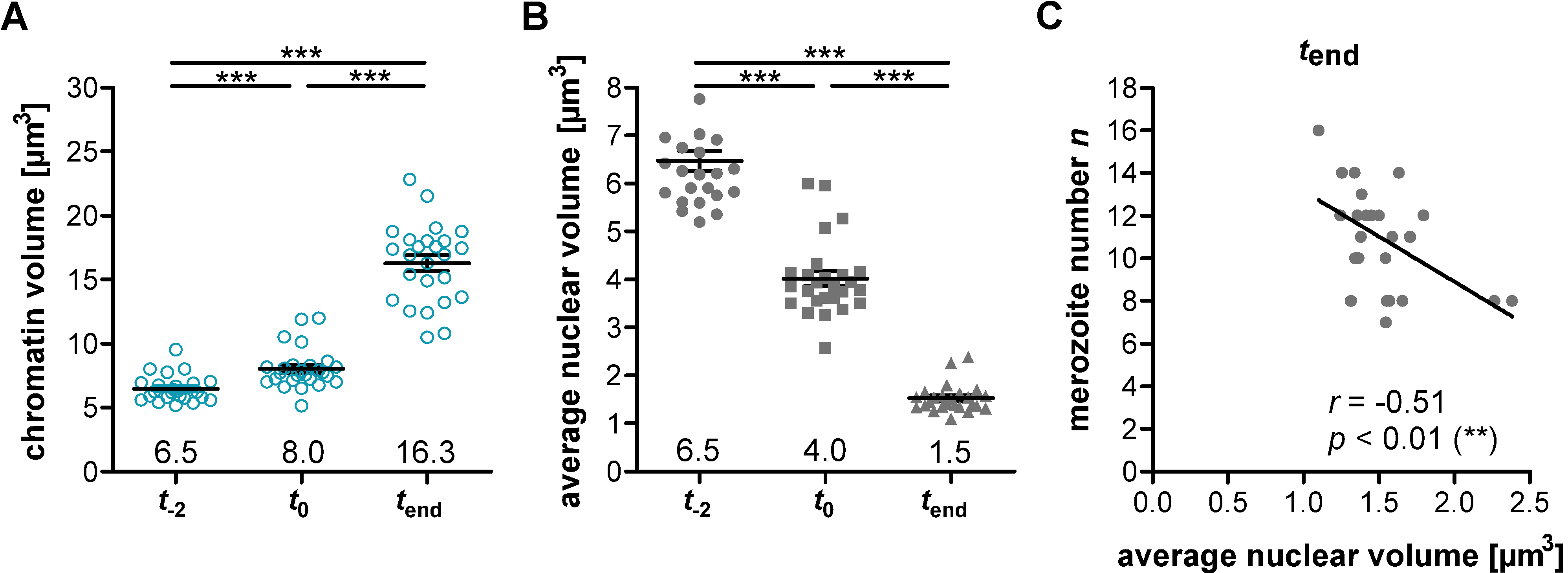

Supplement: S10 Fig — A Chromatin volumes based on H2B-GFP marker for individual cells at three timepoints; pre-schizogony (t−2), schizogony start (t0) and schizogony end (tend). Error bars represent mean and SEM. Statistics: t-test with Welch’s correction. B Average nuclear volume (total chromatin volume divided by nuclear number) for individual cells. C Correlation of merozoite number against average nuclear volume at end of schizogony (tend). Given are Pearson correlation coefficient r and p values. N = 24 for t−2, N = 26 for t0 and N = 25 for tend. (TIF) [file ppat.1011807.s010.tif]

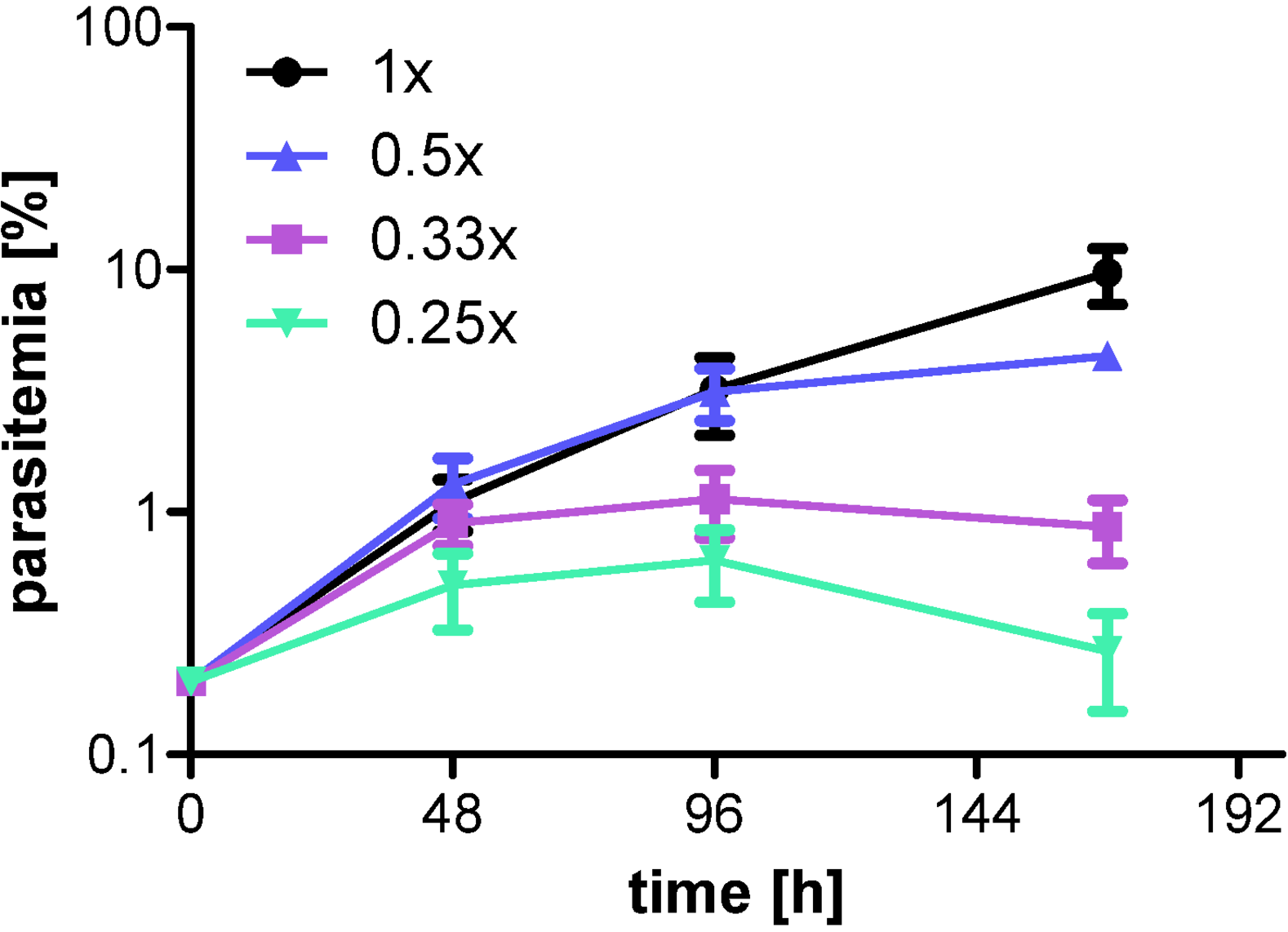

Supplement: S11 Fig — Growth curve with asynchronous parasite cultures with a slight majority of late stages cultivated in normal (1x, black) and diluted (0.5x, blue; 0.33x, purple; 0.25x, green) medium with 0.9% NaCl. Starting parasitemia was set around 0.2%. Medium was changed every 48h and parasitemia was assessed using Giemsa-stained thin blood smears. High parasitemias at the last time point might cause a reduction in growth dynamics for 1x and 0.5x medium, while 0.33x and 0.25x could never be maintained over prolonged time periods. Plotted are mean and SD of three technical replicates. (TIF) [file ppat.1011807.s011.tif]

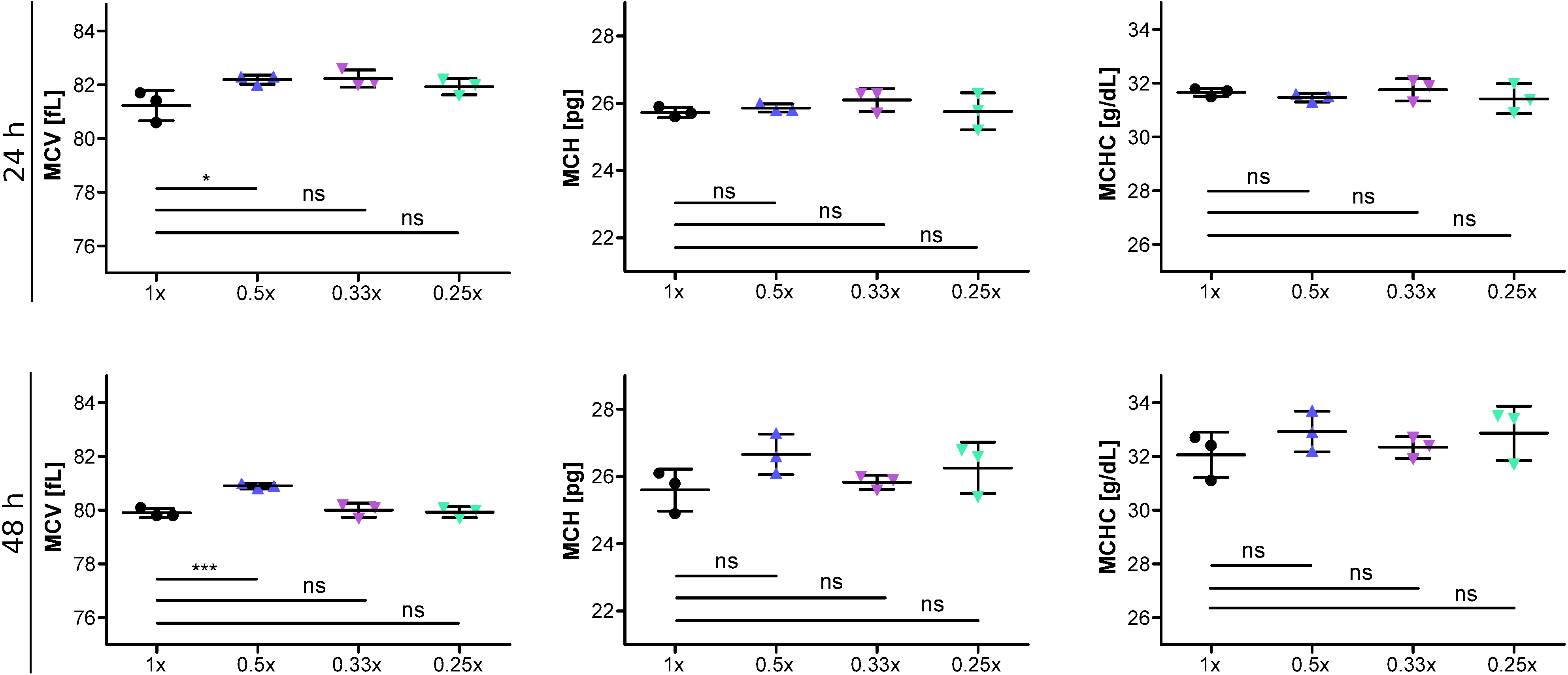

Supplement: S12 Fig — Automated haematology analyses of erythrocytes after 24 and 48 h in different medium dilutions (1x, black; 0.5x, blue; 0.33x, purple; 0.25x, green). Plotted are the indices mean cell volume (MCV) in fL, mean cell hemoglobin (MCH) in pg and mean cell hemoglobin concentration (MCHC) in g/dL. Shown are mean and SD of three technical replicates. (TIF) [file ppat.1011807.s012.tif]

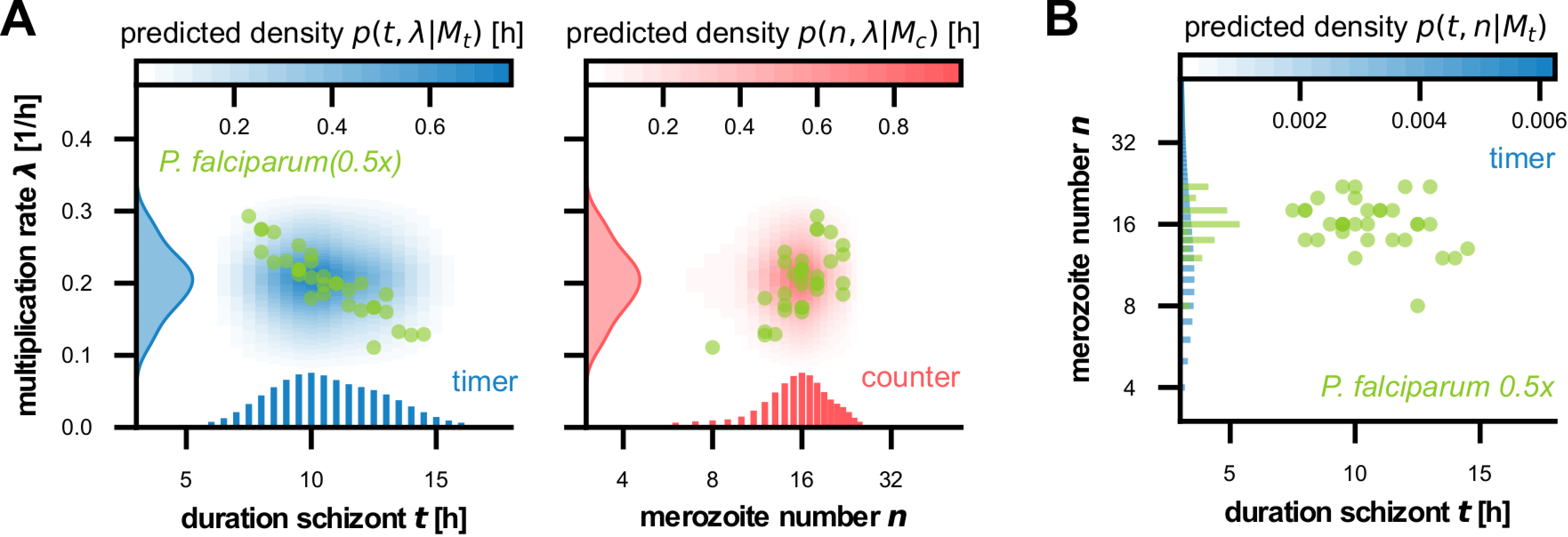

Supplement: S13 Fig — A As (S6C Fig) but for 0.5x medium. B As (S6D Fig, left subpanel) but for 0.5x medium. See also (Fig 6G and 6H). (TIF) [file ppat.1011807.s013.tif]
