# Supplementary material for: Progeny counter mechanism in malaria parasites is linked to extracellular resources
Source: PLoS Pathog. 2023 Dec 5;19(12):e1011807. doi: 10.1371/journal.ppat.1011807 (PMC10723702; doi:10.1371/journal.ppat.1011807)
Supplement: S1 Text — (DOCX) [file ppat.1011807.s019.docx]

**S1 Text. ImageJ macro for cytoplasmic volume determination.**

// ImageJ macro (Java) to segment GFP positive area

// split GFP channel

Exp = // enter experiment date

cell = "" // enter cell number

beforeegress = // add timepoint before egress

rename("GFP");

run("8-bit");

run("Auto Threshold", "method=RenyiEntropy white stack"); // check thresholding with particle analyser outlines

run("Set Scale...", "distance=1 known=0.04 unit=µm");

run("Set Measurements...", "area redirect=None decimal=2");

for (t = 1; t <= beforeegress; t++) { // number of time points

for (z = 1; z <= 20; z++) { // number of z slices

selectWindow("GFP");

Stack.setPosition(3,z,t); //channel,z,t

run("Analyze Particles...", "size=0.05-Infinity show_Masks display exclude slice");

totalarea = 0;

for (n=0; n < nResults; n++){

totalarea += getResult("Area", n);

//volume = totalarea * 0.36;

}

print(t + " " + z + " " + totalarea);

run("Clear Results");

totalarea = 0;

}

}

selectWindow("Log");

saveAs("Text", "/"+Exp+"_"+cell+".txt");
