# Supplementary material for: Progeny counter mechanism in malaria parasites is linked to extracellular resources
Source: PLoS Pathog. 2023 Dec 5;19(12):e1011807. doi: 10.1371/journal.ppat.1011807 (PMC10723702; doi:10.1371/journal.ppat.1011807)
Supplement: Attachment — Submitted filename: 134595_1_rebuttal_2764127_rz3kf1.pdf [file ppat.1011807.s020.pdf]

Full Revision

Review COMMONS

Manuscript number: RC-2023-01894
Corresponding author(s): Julien, Guizetti

1. General Statements

We would like to thank all reviewers for taking the time to evaluate our manuscript fairly and critically. Many helpful suggestions and discussion points were raised. One important group of comments raised concerns whether our proposed timer and counter models were the appropriate conceptual framework to discuss nuclear multiplication in schizogony, whether they were mutually exclusive, and whether other alternatives should be considered. These comments were instrumental for us to uncover some inconsistencies in our previous modeling approach. In the new manuscript, we now define the counter and timer models much more rigorously in the context of Plasmodium cell division. Based on these refined models we now provide a new statistical analysis that goes beyond the previous analysis, significantly improving the statistical support for our conclusions. Details are given in the following individual replies.

All line numbers give below refer to the document with the tracked changes.

2. Point-by-point description of the revisions

Reviewer #1 (Evidence, reproducibility and clarity (Required)):

Summary

Malaria parasites replicating in human red blood cells show a striking diversity in the number of progeny per replication cycle. Variation in progeny number can be seen between different species of malaria parasites, between parasite isolates, even between different cells from the same isolate. To date, we have little understanding of what factors influence progeny number, or how mechanistically it is controlled. In this study, the authors try to define how the mechanism that determines progeny number works. They propose two mechanisms, a 'counter' where progeny number is determined by the measurement of some kind of parasite parameter, and a 'timer' where parasite lifecycle length would be proportional to progeny number. Using a combination of long-term live-cell microscopy and mathematical modelling, the authors find consistent support for a 'counter' mechanism. Support for this mechanism was found using both Plasmodium falciparum, the most prominent human malaria parasite, and P. knowlesi, a zoonotic malaria parasite. Of the parameters measured in this study, the only thing that seemed to predict progeny number was parasite size around the onset of mitosis. The authors also found that during their replication inside red blood cells, malaria parasites drastically increase their nuclear to cytoplasmic ratio, a cellular parameter remains consistent in the vast majority of cell-types studied to date.

Major Comments

It is stated a few times in this study that P. knowlesi has an ~24 hour lifecycle, and while this is the case for in vivo P. knowlesi, it was established in the study when P. knowlesi A1-H1 was adapted to human RBCs

(Moon et al., 2013) that this significantly extended the lifecycle to ~27 hours, which should be made clear in the text. As much of this study revolves around lifecycle length and timing, the authors should consider some of their findings with the context that in vitro adaption can significantly alter lifecycle length.

The reviewer raises an important point that we didn't discuss for *P. knowlesi*. We now mention this directly in the introduction chapter (line 67) and in the discussion (lines 470ff). We are aware that P. knowlesi takes about 27 hours in the lab, which was also communicated by the Moon lab. We now cite relevant studies again in this context. We further address the issue of modified cell cycle time in vitro in the discussion in the sense that absolute values must be taken with caution and the focus of this study is about the relative ratio and correlation between the different cell cycle metrics.

• The dichotomous distinction between 'timer' and 'counter' as mutually exclusive mechanisms seems to be a drastic oversimplification. Considering the drastic variation we see in merozoite number across species, between isolates, and between cells, it seems much more likely that there are factors controlled by both time-sensed and counter-sensed mechanisms that both influence progeny number.

The study of progeny regulation in malaria parasites is very much in the early stages. We can agree that our models are simplifications, as is the case with all models. Our choice of just the two models timer and counter was driven by the number of cellular parameters we measure, i.e., duration of division phase and progeny number. These data essentially allow us to test the two competing models we presented. As we quantify more and more cellular parameters, based on the quantitative live cell imaging protocols established here, we will be able to test more complex cell cycle models. With our current data, we believe more complex models are not warranted.
However, this valuable criticism, in conjunction with related remarks by other reviewers, made us reevaluate the constraints of our model more precisely. We noticed that the criteria used in the previous version in the manuscript contained unnecessary additional assumptions. Briefly, the previous counter model also required that final merozoite number was tightly controlled, while the previous timer model required the growth rate to be tightly controlled. These side assumptions were not made explicit in the manuscript and could bias the support towards one or the other model.
We now improved the modeling approach substantially by removing implicit side assumptions, and clearly defining timer and counter models in terms of their correlations. The refined formulation of the timer posits that between individual parasites the target duration and the nuclear multiplication rate vary in a statistically independent way; while in a counter, target number and nuclear multiplication rate are statistically independent. We now explain this extended analysis in more detail in the introduction (lines 86ff). We also now more clearly state the dichotomous nature of the model (line 488). A new results paragraph (lines 213ff) and an entirely new Fig. 2 (and Fig. S4) contains the model predictions and statistical comparison between the models.
This more rigorous treatment showed that including the variance of the multiplication rate was critical to allow a clean discrimination between the models. Also, with the sole exception of P.knowlesi H2B, where no model was clearly favored (Fig. 2G-H,K), the timer model was found to be inconsistent with the data, while the counter was clearly favored. Our new goodness-of-fit analysis also showed that although the counter is strongly simplified, it produced adequate fits, demonstrating that potential model refinements would need to be justified by new, more extensive data.
It is also important to consider that the degree of variation in merozoite number could rather be an expression of varying growth conditions and does not directly predict which of the proposed models are true. For instance, a counter where the target merozoite number varies strongly depending on growth conditions, would be consistent with all available data. It is an interesting question for future work whether a counter would indeed describe growth across different isolates.

The biological reality of growth regulation is certainly complex, and the counter model will likely need to be refined in the future, which we acknowledge in a corresponding statement in the discussion (lines 491ff). Nevertheless, we find it encouraging that a simple model can explain the vast majority of our data very well.

Additionally, the only parasite parameter measured in this study, size at time of first nuclear division, explained only a small proportion of the variance observed in merozoite number.

It is indeed the case that amongst the measured parasite parameters i.e. schizont stage duration, nuclear volume, and cell size we only found the latter to correlate with the final progeny number. We did not aim to imply that all variation in progeny number is explained by cell size. It is likely that a putative counter relies on a set of factors, which are somehow linked to cell size. In addition, intrinsic stochasticity in nuclear growth is likely to contribute to final merozoite number variability, which is included in our models via a variable growth rate. Defining the actual limiting factor or combination of factors will be an exciting challenge for the future studies building on this one.

• For modelling of a timer-based mechanism, the designation of t0 is subjective. The authors chose the time of first nuclear division as their t0. It is possible that a timer-based mechanism could not be supported based on this model the chosen t0 differs from when the "parasite's timer" starts. For example, t could also have been designated as the time from merozoite invasion (t0) to egress (tend). It would be unreasonable to suggest the authors repeat experiments with a longer time-frame to address this, but this possibility should be discussed as a limitation of the model. It may also be possible to develop a different model where t0 = merozoite invasion and tend = egress, and test this model against the data already collected in this study.

This is a valid point. We indeed, considered the time point of invasion as the other relevant time point in the IDC for a possible timer. Due to necessary compromises in imaging protocols between acquisition length, temporal, and spatial resolution we have not been able yet to combine full-length IDC measurements with quantification of progeny number. Given the choice, however, between time point of invasion and the onset of nuclear division as starting point for a potential timer we would still favor the latter: An argument can be made that a timer that regulates offspring number would be more accurate when activated at the moment of the relevant cellular events rather than “running” for a very prolonged growth phase before any “decision” concerning parasite replication. We are still convinced that the entry into the schizont stage, which we analyze here, marks an important cell cycle transition point that has been highlighted in many different studies. As suggested, we now discuss the limitations of our selection of t0 in the text (lines 146ff).

• The calculation of the multiplication rate is confusingly defined. In Figure 1 it is stated that it is "...based on t and n", which would imply that the multiplication rate is the number of merozoites formed per hour of schizogony, which would give an average value of ~2 for P. falciparum and ~1.5 for P. knowlesi. The averages rate values shown, however, are in the range of 0.15-3. The authors should clarify how these values were determined.

Thank you for pointing out the need for more clarity. Since the nuclear multiplication, similar to e.g. cell population growth, follows an exponential law, the multiplication rate used (lambda) is in fact a logarithmic growth rate. Therefore, it occurs in the exponent (not as a coefficient) in the exponential growth function ($n(t) = 2\ e^{\lambda t}$), which explains the range. We now mention this more explicitly in the results (lines 163ff).

• In Figure 2, the time from tend until egress is calculated, and this is interpreted as the time required for segmentation. In the Rudlaff et al., 2020 study cited in this paper, it is shown that segmentation starts before the final round of nuclear divisions are complete. Considering this, the time from tend until egress is not an

appropriate proxy for segmentation time. The authors should consider rewording to something akin to "time from final nuclear division until egress" to more accurately reflect these data.

Thank you for indicating our imprecise use of the nomenclature. Indeed, some essential segmentation-associated structures such as rhoptries and subpellicular microtubules are clearly forming before the last division. We were referring to “segmentation” as the time window where actual ingression of the plasma membrane occurs between nuclei with the concurrent formation of more prominent IMC-associated sub-pellicular microtubules between nuclei (as in Fig. 1A last panel). We can, however, agree that consistently using the term “merozoite formation” is more adequate here. We have now corrected the terminology according to the suggestions of the reviewer (lines 271ff).

• There is a significant discrepancy between the data in Figure 5 and Supplementary Figure 8. In Supplementary Figure 8, the authors establish that culturing parasites in media diluted 0.5x has a marginal effect on parasite growth, with no discernible change in parasitaemia over 96 hours. By contrast, in Figure 5a the parasitaemia of parasites cultured in 0.5x diluted media is approximately 5-fold lower than those in 1x media. The authors should explain the significant discrepancy between these results.

The reviewer correctly points out a difference in parasitaemia between two parasite culture experiments, shown in Figs 5a (now 6A) and S8 (now S11), respectively. There were several differences in the experimental setup used in the two experiments that could explain this discrepancy. In Fig. 5a the parasites were synchronized to early ring stages while in Fig. S8 we used asynchronous cultures (maybe with a slight majority of late stages). One could speculate that by the time the synchronized ring stage culture reached egress the effect of nutrient depletion, which started at t = 0 h is more pronounced. This effect could have been exacerbated by the more frequent media change of 24 h in Fig. 5a vs 48h in Fig. S8. Lastly, the starting parasitemia was differently set being higher at around 0.5% in the Fig. 5a while only 0.2% in Fig. S8. Possibly a lack of nutrient is “felt less” by the culture at lower parasitemias. Generally, in Fig. S8 we were more focused on highlighting the difference between 1x/0.5x and the more diluted conditions on the long-term culture and to show that continuous culture is actually possible in 0.5x medium. We have now expanded the legends to highlight those differences more clearly.

• In Supplementary Figure 4, the mask on the cell at t0 shows two distinct objects, but it seems very unlikely that they are two distinct nuclei as they vary approximately 5-fold in diameter. The authors should provide more detail on how their masking was performed for their volumetric analysis. Specifically, whether size thresholds were also applied during object detection.

Thank you for requesting clarification here. Fig S4 (now S7) shows only one z-slice (not a projection) of the entire image stack, to illustrate how the thresholding approach was performed on every single image slice. The two objects in the shown cell are indeed two nuclei, but because they are not in the same z-plane appear to be of different size. In particular, only a slice of the upper part of the nucleus on the lower right is visible in the shown slice. Throughout the study, volume determination was realized by adding up the individual slices, as is explained in detail in the Materials and Methods sections. We have now added a more explanation in the figure legend to clarify the procedure.

Minor Comments

• Line 45-48 mentions that merozoite number influences growth rate and virulence, but the corresponding reference (Mancio-Silva et al., 2013) only discusses the relationship between merozoite number and growth rate, not virulence.

We thank the reviewer for requesting this distinction. Merozoite number and virulence have not been correlated in vivo so far. Certainly, because one can't retrieve late-stage *P. falciparum* parasites from patients, but maybe partly because merozoite number has not gotten significant attention as a metric in the previous decades. Even if merozoite number is intuitively connected to growth rate which might causes higher parasitemia which is in turn linked to more severe disease outcome it is important to emphasize that those are certainly not equivalent. We have therefore removed the statement about virulence (line 48).

• Line 59 states that a 48 hour lifecycle is a baseline from which in vitro cultured parasites deviate. Clinical isolates also show variation in lifecycle length and so it is more accurate to just say that 48 hours is an average, rather than a baseline.

The word "baseline" has been changed to "average" (line 61).

• Line 63 cites a study for the lifecycle length of P. knowlesi (Lee et al., 2022), but there seems to be no mention of lifecycle length in this reference

This reference was meant to serve as an introductory review article to research in *P. knowlesi*. Actually, to the knowledge of the authors, there is no study presenting quantitative data showing that the in vitro cycle of P. knowlesi is actually around 27 h. Our lab experience is however coherent with a 27 h cycle, which was confirmed by personal communication by the Moon lab. We now also cite in the next sentence the inaugural *P. knowlesi* adaptation publication (Moon et al. 2013) showing some time course data indicating the duration of the IDC to be around ~27h (lines 67ff).

• If I am interpreting Figure 3B correctly, this is essentially a paired analysis where the same erythrocytes are measured twice, once at t0 and once at tend. If this is the case, this data may be better represented with lines that connect the t0 and tend values.

Yes, these are the same erythrocytes measured twice. We have modified Figure 3 (now Fig. 4) accordingly.

• Figure 3A seems to imply that to calculate diameter of the erythrocytes, three measurements were made and averaged for each cell. I think this is a nice way to get a more accurate erythrocyte diameter, but if this is the case, it should be specified in the figure legend or methods.

This is already described in the figure legend (line 305).

• In Figure 4I it is shown that in P. falciparum merozoite number doesn't correlate with nucleus size, but for P. knowlesi in Supplementary Figure 7c, a significant anticorrelation is observed. The authors should state this in the text and discuss this discrepancy.

Contrary to all other graphs, visual inspection of the distribution of data points in Fig. S10C shows that it contains two outlier data points at the bottom right. Those two specific points are also responsible for the significant anticorrelation. We did not filter or remove any quantification results but also didn't have sufficient confidence in this data distribution (which is further based on the segmentation of the Histone2B not on an NLS mCherry signal) to make substantial claims about anticorrelation. Because we considered it informative we still decided to show it in the supplements. We now briefly mention the issues with the data set and its interpretation in the text (lines 350ff).

Review COMMONS

• The authors show that merozoite number roughly correlates with cell size at t0 but it would be interesting to see whether cell size at tend also corresponds with cell size at t0. This might help answer whether the cell is larger because it has more merozoites, or whether it has more merozoites because it is larger.

Plotting parasite cell volume at t0 against cell volume at tend (as well as between t-2 and tend) indeed shows a positive correlation (see below). While it is an interesting thought we concluded after some discussion that no convincing causal relationship between cell size and merozoite number can be inferred based on this analysis. Since we consider the possible statement that cells that are bigger in the beginning are also bigger in the end unavailing, we decided not to include the data.

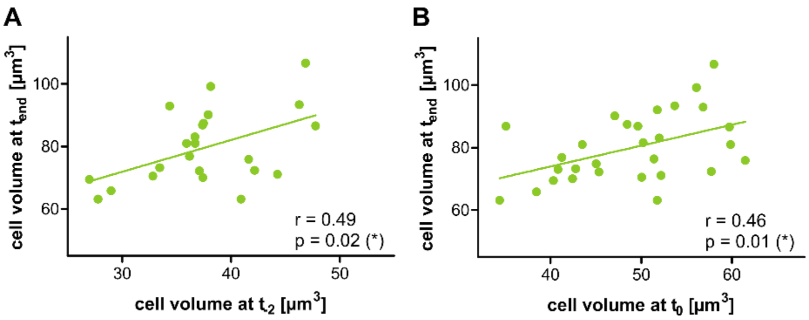

• I don't feel that "nearly identical" is an appropriate summary of erythrocyte indices in Supplementary Figure 9, considering there is a statistically significant increase in mean cell volume. I think it is unlikely that this change is consequential, and performing these haematology analyses is a nice quality control step, but this change should be stated in the text.

In the modified text we now express the significant change in MCV in terms of percentage, which is around 1.2% (line 381).

• In Supplementary Figure 8, parasitaemia only increases ~2-fold compared to >5-fold the previous two cycles. It seems likely that at the final timepoint on this graph the parasites are starting to crash, and therefore it may be best to end the graph with the 96 hour timepoint.

The reviewer suggests that cultures at those parasitemias might not be in perfect health. Our Giemsa stains did not show signs of an unhealthy culture and kept growing. It was, however, important for us to show that cultures can be maintained in culture over a prolonged period of time in 0.5x medium, even when resulting in reduced growth, while this was not possible with lower dilutions. Therefore, we would like to keep the data point. We have added a cautionary comment in the legend.

• The error bars in Figure 5C aren't easily visible, moving them in front of the datapoints may help their visibility.

Error bars were moved in front of the data points.

• In Figure 6D & E, the y-axis labels should be changed to whole integers as all the values in the graph are whole numbers.

We have changed the y-axis labels accordingly.

• My interpretation of Figure 6 C-E, is that these are the same cells measured at three time points (t-2, t0 and tend). If this is the case, 6C is missing the cell that has a merozoite number of 8, which is presumably why the y-axes are not equalised for the three graphs.

It is correct that the same cells are displayed in all three plots, with the exceptions of three cells in 6C (for the timepoint t-2), which are missing for the following reasons: 1) it was not possible to determine the volume at this respective timepoint due to technical issues or 2) the cell was already just before t0 at the start of the movie so that t-2 had already passed. We now note this in the figure legend and have also equalized the y-axes (now Fig. 7C-E).

Reviewer #1 (Significance (Required)):

In the asexual blood-stage of their lifecycle, malaria parasites replicate through a process called schizogony. During schizogony an initially mononucleated parasite undergoes multiple asynchronous rounds of mitosis followed by nuclear division without cytokinesis, producing a variable number of daughter nuclei. Parasites then undergo a specialised cytokinesis, termed segmentation to where nuclei are packaged into merozoites that go on to invade new host cells. While nucleus, and therefore merozoite, number are known to be varied between cells, across isolates, and across species, little is known about the mechanisms regulating merozoite number. In this study, the authors use live-cell microscopy to understand how parasites determine their progeny number. They suggest that parasites regulate their progeny number using a 'counter' mechanism, which would respond to the size or concentration of a cellular parameter, as opposed to a 'timer' mechanism. Long-term live-cell microscopy experiments using malaria parasites are extremely technically challenging, and the authors should be commended for their efforts in this regard. While I agree that the data generated from these experiments are technically sound, I have some reservations expressed above about the interpretation of some of these results. I would strongly encourage the authors to consider rewording some of their interpretations taking into account some of the caveats listed above. I would also consider fitting/testing an additional mathematical model where the time-frame proposed for the 'timer' mechanism begins following merozoite invasion.

We thank the reviewer for the appreciation of our work and hope we have sufficiently reworked the manuscript based on the comments listed above. Furthermore, we think the improved model statement and analysis improves the clarity of our conclusions. Indeed, we would like to test additional models including the full IDC once, as mentioned above, we are technically able to generate these data.

This work is of specific interest to anybody who grows malaria parasites, as the dynamics of their growth is obviously important to understand. Further, this work is of interest more generally to cell biologists who study the regulation of progeny number or cell size. I have no experience with the application of mathematical modelling to understand biological systems, and so I cannot comment on the interest of this work to that field.

Review COMMONS

Reviewer #2 (Evidence, reproducibility and clarity (Required)):

This is a solid study that further characterises the dynamics of nuclear division in Plasmodium falciparum and P. knowlesi. Of two, among potentially several, models for how the number of daughter nuclei, and thus parasites - (called merozoites in this genus), are one that posits nuclei divide until a fixed timer ends, and one that posits that nuclei divide to reach a fixed number that is defined by a cellular counter. I find some practical difficulties in definitive measurement of either model, one issue with the former is that experimental definition of the start of the timer is problematic - we may define the starter's gun (eg by the first nuclear division) but it isn't necessary that the cell is using that same start time.

We are pleased that the Reviewer found our study 'solid'. Concerning the timer model, we agree that the selection of the starting point is a critical aspect of this study, as also Reviewer 1 pointed out. We selected this particular "t0" because the entry into the mitotic phase marks an important cell cycle transition. Several studies have suggested a "schizogony entry checkpoint" might be active just before (Matthews *et al*, 2018; Voß *et al*, 2023; van Biljon *et al*, 2018; McLean & Jacobs-Lorena, 2020). Once cells are committed to the schizont stage they are less responsive to stimuli. Alternatively, the timepoint of erythrocyte invasion could be a legitimate starting point. Due to necessary compromises in our imaging protocol between acquisition length, temporal, and spatial resolution we have not been able yet to combine full-length IDC measurements with quantification of progeny number, and therefore we leave exploration of an earlier timer start for future work. Within the confines of the model comparison in the current study, we think the selected t0 is already highly informative. We now explain the selection and limitations more explicitly in the text (line 144ff).

Additionally, as the authors confirm here, being sure when that first nuclear division has occurred is particularly tricky with Plasmodium parasites, in part because the first few nuclei seem to clump together, preventing one from unambiguously calibrating the first division.

The Reviewer is concerned about difficulties with precise reporting of the time point of first nuclear division. We suspect there was a misunderstanding here. In the text (line 137) we had written the following: "Although separating individual nuclei after the first two rounds of division was challenging due to their spatial proximity, the improvements in resolution and 3D image analysis allowed us to count the final number of nuclei routinely and reliably at the transition into the segmenter stage."
To clarify, when analyzing 3D image stacks produced by the LSM900 Airyscan the first nuclear division can consistently and unambiguously be detected. In anaphase the nuclei are pushed apart quite substantially before getting a bit closer together afterwards (see e.g. Fig. 1B and C). Hence the precision of the detection is only limited by the 30 min interval of the time lapse. Later, at the four nuclei stage, crowding makes distinction more difficult. In the final segmenter stage, the reorganization and condensation of nuclei makes reliable counting possible again. We have now reformulated the quoted sentence for more clarity (lines 137ff).

Furthermore, getting decent replicate numbers is hard because of the difficulties of time lapse microscopy, and most Plasmodium studies (including this one) suffer from low enough numbers that it isn't always clear whether the numbers support one model over another.

The reviewer points out the difficulty of obtaining enough replicates in Plasmodium time-lapse studies. We agree that depending on technology, sufficient replicates can be challenging. In the present study we obtained Ns between 25 and 35 for all conditions in *P. falciparum* and *P. knowlesi* from three independent replicas. To gain confidence in the conclusions from a limited, but not austere, data, it is essential to 1)

reduce model complexity to a minimum and 2) perform stringent statistical analysis including accounting for small-sample variation. Motivated by this concern of the Reviewer and a similar point raised by Reviewer 1, we have revisited our modeling approach in the revised manuscript. This led us to a corrected, more rigorous definition of what precisely we mean by 'counter' and 'timer' models: The timer posits that between individual parasites the target duration and the nuclear multiplication rate and vary in a statistically independent way, while in a counter target number and nuclear multiplication rate are statistically independent. With no further adjustable parameters, the two models are thus both mutually exclusive and minimal. Although biological reality is likely to be more complex, we feel that these minimal models are adequate for the amount and resolution of our current, state-of-the art data. The general result remained the same: The counter model is strongly preferred in almost all our experiments data (new Fig. 2), with the sole exception of *P. knowlesi* H2B, where indeed more data may be needed to come to a clear conclusion. Furthermore, we have taken care to scrutinize these conclusions accounting for goodness-of-fit for the respective sample size N. This analysis showed, surprisingly, that the counter model was sufficient to account for the data: the real dataset was as similar to the counter prediction as synthetic, counter-generated data. We hope that this improved statistical analysis can help the reader judge the robustness of our conclusions.

Nonetheless, several recent studies, particularly a study from the same institute (Klaus et al., 2022) employing timelapse imaging of nuclei, and timing the nuclear division of parasites, finds poor correlation between the duration of "schizogeny" (although perhaps using a different definition to the one used by the parasite) and the final number or merozoites. They therefore argue that there is poor evidence for a timer, and conclude by elimination that a counter must exist instead. A review by some of the authors of that study and some of this current study (Voß et al 2023), also concludes that the data from Klaus and colleagues "strongly support" a counter model. This current study also concludes that a counter model controls final nuclear/merozoite number in P. falciparum and P. knowlesi. This much at least is not particularly novel given the recent work on this topic, although the addition of the P. knowlesi data is interesting and consistent with the prior P. falciparum work.

Our present work, indeed, does confirm the previous report of a counter over a timer, through a more targeted approach. While Klaus et al. used timing data of first nuclear cycle vs. the full duration, we now provide, thanks to an improvement microscopy setup and protocol, simultaneous measurements of timing and final progeny number, i.e. counting of merozoites/nuclei. While the preference for a counter model is not fundamentally novel, the additional information that the counter model holds in different strains, conditions and species is, in our opinion, not trivial and points to some degree of evolutionary conservation. We also demonstrate here that the counter model is not only preferred over the timer, it also fits the data adequately, so that it can be considered 'correct' at this level of complexity. Another, possibly more important, value of this study lies in the quantitative and time-resolved assessment of multiple important parasite metrics such a cell volume and nuclear volume together with merozoite number at the single cell level. Although descriptive, this has not been achieved in Plasmodium until now.

As above, the authors concede that it is difficult to determine with strong confidence when the first nuclear division has occurred, so it may well be that there is substantial noisiness in the time that they define schizogeny to commence. If that were the case, this would contribute to the poor correlation observed between schizogeny duration and number of merozoites produced, so this could be an important confounding experimental factor. This deserves some more discussion by the authors.

Concerning the confidence with which we identify the first nuclear division we could hopefully clarify in the section above that our precision is only limited by the time resolution of the acquired time-lapse. Therefore, the uncertainty about the start time is not particularly high, and moreover, can expected to affect

timer and counter (via the growth rate) to a similar degree. We see no unfair advantage for the counter for this reason.

Alternative methods to count absolute DNA content (rather than trying to count individual nuclei) might be useful ways of independently confirming this phenomenon. Alternative possibilities for what constitutes the "start" of a possible timer are also warranted - it could be for example, the first division of one of the other organelles.

This is an interesting suggestion. Next generation fluorogenic DNA dyes have been used by us and the Ganter group (Simon et al. 2021, Klaus et al. 2022, Wenz et l. 2023) to assess DNA content of single cells over time. Our experience shows that there are some caveats to using these Hoechst based dyes, some of which we discussed in the aforementioned publications. While they allow some reasonable absolute quantification of DNA content for the very first S-Phase (and subsequent nuclear division), in later stages only relative quantification can be achieved. One underlying reason is the apparent increase of dye permeability, and therefore higher intensity, at late schizont stages. This issue is exacerbated by the asynchronous DNA replication of multiple nuclei. Further, nuclear division itself can be delayed or even inhibited when increasing the concentration of the dye, which suggest an impact on cell physiology (well documented for Hoechst based dyes in other organisms). When reaching the segmenter stage, the resulting variance in fluorescent intensity would make it challenging to assign a reliable number of nuclei required for analysis, a problem that does not occur when counting individual nuclei. Taken together, unfortunately, all these confounding factors make DNA content analysis in live single cells for the entire schizont stage unachievable at this point.

These and previous authors in any case conclude that a counter model must exist through exclusion of a timer model. I am less convinced that the evidence discounting the timer is conclusive, and that a straight counter model is the only alternative. Indeed I am unconvinced by the suitability of this strictly dichotomous two-model system to categorise the division of unicellular eukaryotes, and these theories are not universally held to be sufficient to describe division.

We thank the Reviewer for this insightful comment. As already detailed above, we have clarified and corrected our model definitions in the revised manuscript. Further, we want to make the important distinction between organisms, including unicellular ones that undergo binary fission and the ones like Plasmodium that use schizogony. Our model, although inspired by model organisms, is tailored to a multinucleated division mechanism, and clearly defined within those boundaries. The timer and counter models we consider are defined by their correlation structures. They are at two extremes of a continuum of models which could be characterized, for instance, by the ratio of correlations (growth rate - nuclear number) vs. (growth rate – duration) as an additional parameter. As the reviewer points out, excluding the timer model is not equivalent to proving the counter model, and indeed a partially correlated model, or a more complex model entirely, could yield a better fit. However, within the realm of models without additional parameters, and which are testable with the available data, only timer and counter remain, as different timer start points are not experimentally accessible. Importantly and somewhat surprisingly, the counter model also gave a fit that is as good as can be reasonably expected for the experimental sample size (new Fig. 2). So, we maintain that within the current experimental constraints, the counter model is the only viable option for almost all our tested conditions. The observation that in H2B-GFP expressing *P. knowlesi* parasites no clear distinction can be made between the models, indeed, suggest that the reality of multiplication rate regulation is more complex and may be limited by different constraints in different growth regimes. We now state these limitations and the room for further model adjustments with more data in the Discussion section.

Nonetheless, if a counter exists, what is being counted that determines the final number? The authors consider that this might be a physical object or resource inside the parasite, or an extrinsic/extracellular resource. They investigate this by comparing the final cell number to a number of factors. First, the authors investigate the size of the RBC (by musing the diameter as an indicator)- little information is given about the source of the blood used, but it appears to be from a single donor of unknown age, who has approximately typical variance in RBC diameter (at least, after manipulation and storage). The authors observe little correlation between these variables.

We share the curiosity of the reviewer about what might be “counted” by the parasite. This shall be the subject of future studies, and our present study provides the necessary basis for asking this question and defines a framework to investigate it. Concerning the size of the host cell, the blood used was from a different donor for each of the replicas, which we now specify in the figure legend (line 302). No significant difference between the RBC diameters between the donors was observed. A correlation between RBC diameter and progeny number was indeed not observed.

Second the authors measure parasite size at the onset of schizogeny, and find that bigger parasites result in more daughter merozoites early in schizogeny (perhaps not surprising, given the earlier mentioned technical problems with measuring the first few steps of schizogeny), but that this different initial cell size doesn't result in a different final merozoite number, or as they describe it "not quite significant anymore". Previous p values were taken as cause for rejecting the timer hypothesis and the timer model. In this case the authors instead interpret the data as suggesting "that the setting of the counter might correlate with parasite cell size". This is inconsistent statistical and analytical handling, and highlights the earlier potential pitfall of rejecting timer-based models based on not gathering data that statistically show a correlation. This needs reworking to highlight that these data are inherently noisy, difficult to measure accurately, and aren't necessarily going strongly reveal a trend even where one biologically exists, and that this ought not be used as grounds for confident rejection of a model.

The Reviewer raises concerns about the consistency of the statistical interpretation of our data. We care deeply about the well-foundedness of our conclusions and hope to eliminate these concerns in the following. First, we hope that the issue about the “technical problems” in measuring the first division has been solved in our response to previous comments. Next, to clarify an apparent misunderstanding: As stated in the text (lines 329ff) and shown in now Fig. 5D-E, cell size at onset of nuclear division or 2 hours prior does significantly correlate with final merozoite number. The lack of significant p-value (0.08) only pertains to the correlation of cell size at the end of the schizont stage (t_{end}) with merozoite number (now Fig. 5F). We have removed the unfortunate wording “not quite significant anymore” in that context. Finally, regarding potential mechanisms, a potential counter must be set before the first nuclear division is completed because only that way it can be set independent of the speed of nuclear multiplication. This observation gives the statistically significant correlation of volume at the onset of division and progeny number its relevance. We have reformulated the marked sentence for more clarity (lines 331ff). Furthermore, we point out that our rejection of the timer is now based on a revisited statistical analysis (Fig. 2), which is no longer based on a simple correlation between final number and duration, as detailed above.

Finally, the authors grow the parasites in dilute media, and find that they produce fewer daughter parasites. This is anecdotally unsurprising, as most Plasmodium laboratories are aware that sub-optimal growth conditions result in less healthy schizonts with fewer viable merozoites (and lower magnitudes of single-cycle expansion), but is nonetheless an important result that highlights explicitly how much this occurs in the specific conditions of dilute media. Given the lack of investigation of exactly which nutrient, carbon source, or combination thereof leads to the reduced merozoite number, it is unclear if or how much this is

relevant to the scenario of a natural infection and realistic levels of that nutrient in a human or primate parasite environment.

As rightfully pointed out by the reviewer suboptimal growth conditions affecting parasite growth and multiplication rate have been shown in many instances. The number of studies that actually quantify a reduction in merozoite number under different growth conditions is certainly much lower (Brancucci et al. 2017 (lipids), Mancio-Silva et al. 2017 (calorie-restriction in mice), Tinto-Font et al. 2022 (temperature) come to mind). What our study adds to this body of literature is to which extent duration of the schizont stage and cell volume are affected in relation to progeny number at the single cell level. Importantly, we wanted to test whether the counter model still holds under these more adverse conditions, which we found to be the case. Along the lines of the work on calorie restriction and the likely implication of isoleucine in the process investigated in the laboratory of Maria Mota, it will be exciting to identify a "limiting factor" in future studies. Indeed, any study done in complete RPMI culture medium can be questioned regarding its physiological relevance and we added a sentence addressing this aspect in the discussion (lines 514ff). Yet, our medium dilution experiments suggest that at least to some degree an extracellular resource is implicated, which makes sense from a biological function point-of-view.

Minor issues

The manuscript confuses the terms "less" and "fewer". Fewer should be used for countable nouns (fewer daughter cells, fewer nuclei, fewer merozoites), less for uncountable nouns (e.g. less speed, less volume).

Thank you for pointing this out. The words have been replaced accordingly.

I didn't understand lines 93-95; "This excluded a timer and thereby confirmed a counter as the mechanism regulating termination of nuclear multiplication (Klaus et al., 2022). A direct correlation between duration of schizont stage and merozoite number is, however, still missing." If I understand the first sentence concludes that there ought not be a direct correlation between schizont duration and merozoite number, but the second sentence, says that that correlation is "however" missing. Isn't this expected? Perhaps reword for clarity?

Thank you for requesting clarification here. The exclusion of the timer by Klaus et al. 2022 was based on the correlation between duration of the first nuclear division cycle and the total duration of all nuclear replication phases. At no point did Klaus et al. count merozoites in live single cells, which was mainly due to lower spatial resolution of their images (M. Ganter, personal communication). Therefore, they could not directly assess the relation between progeny number and schizont stage duration, which we now report for the first time. The sentence was supposed to convey that this type of data was missing and was now reformulated for more clarity (line 114).

Lines 104
"We further uncover that throughout schizogony P. falciparum infringes on the otherwise ubiquitously constant N/C-ratio (Cantwell and Nurse, 2019)" This seems obvious to me, and not something uncovered by this study. In most of the numerous apicomplexans that divide by endoschizogeny, the cells achieve a near final size considerably before the final rounds of nuclear division so the N/C ratio must not remain constant - this is a direct corollary of many previous descriptions and not a novel finding of this study, and this claim here should be made more modest.

We understand the point raised by the reviewer but still think that our claim is justified due to several aspects. There are examples of eukaryotic cells that undergo multinucleated stages during division were the

N/C-ratio is constant (Dundon et al. 2016, Cantwell and Nurse, 2019), while we are not aware of any counter-example in the literature. Studies have also shown that e.g. certain mutant yeast that fail to undergo cytokinesis will increase their volume by factor of up to 16 alongside the still replicating and growing nucleus maintain the N/C-ratio (Neumann et al. 2007, Jorgensen et al. 2007). This demonstrates the tremendous plasticity that cells can reveal with respect to nucleus and cell size regulation. Until the contrary was shown, it was conceivable that nuclear compaction, which does occur (Fig. 5H), compensates for the increase in nuclear number while the cell volume is only increasing slightly. Importantly, we are not aware of any literature where nuclear volume has been quantified for blood stage Plasmodium. Cell volume quantifications remain limited to modelling and the study by Waldecker et al., which provides a few datapoints throughout the IDC. Whether this finding is expected or not, formally speaking, our claim is justified, but for more clarity we replace "uncover" with "demonstrate". We also introduce the N/C-ratio as cellular parameter in *P. falciparum* pointing out another divergent aspect of its biology and might in the future understand the functional implication of this usually constant ratio, which is still unclear.

Dundon SE, Chang SS, Kumar A, Occhipinti P, Shroff H, Roper M, Gladfelter AS. Clustered nuclei maintain autonomy and nucleocytoplasmic ratio control in a syncytium. Mol Biol Cell. 2016 Jul 1;27(13):2000-7.

Neumann FR, and Nurse P. Nuclear size control in fission yeast. J. Cell Biol. 2007; 179: 593–600. pmid:17998401

Jorgensen P, Edgington NP, Schneider BL, Rupeš I, Tyers M & Futcher B Molecular Biology of the Cell 18 (2007) The size of the nucleus increases as yeast cells grow.

Helena Cantwell, Paul Nurse; A homeostatic mechanism rapidly corrects aberrant nucleocytoplasmic ratios maintaining nuclear size in fission yeast. *J Cell Sci*; 132 (22)

I lack specialist statistical knowledge to comment on the statistical analyses performed on the correlation data, and in particular, whether the high p values for t-Tests for correlation are sufficient to support the argument that there is not a correlation, and whether these observations are sufficiently powered to robustly test that hypothesis.

We are confident that our reworked model analysis, as explained above, now sufficiently supports our hypotheses.

Reviewer #2 (Significance (Required)):

The manuscript purports to find a counting mechanism that determines parasite merozoite numbers, and that this coutner is set by an externally provided and diffusible resource. Many nutrients are in excess in normal culture media, but not all. If that counted nutrient(s) were normally in excess in the bloodstream, it could hardly be said to be the factor that is counted and that therefore defines merozoite number. Conversely, if the amount of that nutrient were increased in normal media, would parasites make even more merozoites? Further, if the "counted" item is a freely diffusible compound in the media, it should be equally accessible to each parasite in a culture condition, and isn't a reasonable explanation for the variable merozoite numbers in the normal media conditions. To me, it is unsurprising that parasites that are healthy and well fed are able to produce more merozoites, but I don't see this as being the same as support for a counter model where the parasite senses and counts a set number of merozoites to produce in response to a specific external counter. I think the shoehorning of this phenomenon into a paradigm used to describe some other eukaryotes may not be appropriate, and that the rejection of one overly simplistic timer model should not automatically lead to us dichotomously accepting a simple counter method as the alternative. The authors need to do more to either identify a countable input whose gradual increase leads to a predictable and gradual increase in merozoite number, to show that they do use a counter, or provide

substantially more caveats to their argument that the parasites are using a counter based on an externally provided resource to determine merozoite number.

The reviewer comments on the feasibility of a counter mechanism based on an externally provided and diffusible resource. In fact this is a limited view of how a counter may arise and not the one we subscribe to. Rather, while a resource may be diffusible in the medium, it would need to be consumed during schizogony, and insufficiently replenished, in order to enable counting by dilution in the host cell. Furthermore, the reviewer has doubts that the fact that “healthy and well fed […] produce more merozoites” implies “support for a counter model”. We fully agree, and we argue in the manuscript that it is the correlations between schizogony durations and merozoite counts that support a counter model.
As we have argued above, the two alternative models we consider are inspired by paradigm from other eukaryotes, but their definitions in the present context are simple enough for them to be considered natural minimal models of schizogony. As the simplest imaginable phenomenological models of multiplication control, we find it natural to compare them, and we hope our new introductory section introduces them appropriately now. Naturally, we hope to expand on this simple model in the future and identify more precisely the limiting resources and describe a more direct response.

Audience - relatively specialised - likely interested audience would combine apicomplexan cell biologists, as well as theorists of cell division mechanism

Advance - limited - confirms phenomenon also described by other researchers in their institute, and extends to another related organism.

We would like to add that the present data are the first quantitative joint measurements of schizogony dynamics and outcome in *P.falciparum* and *knowlesi*. They allowed for the first time a direct correlation of duration and merozoite number, thereby accessing the question of growth control head on. Further they provide a quantitative reference of several key cellular parameters for anybody studying asexual blood stage parasites.

Reviewer #3 (Evidence, reproducibility and clarity (Required)):

Summary:
Stürmer and colleagues used super-resolution time-lapse microscopy to probe the mechanism regulating the number of merozoites produced by a single cell in Plasmodium falciparum and P. knowlesi. The authors conclude the followings-
a. P. knowlesi has similar duration of schizont stage to P. falciparum, although having a 24 h intraerythrocytic developmental cycle (IDC) to 48 h of P. falciparum.
b. Nuclear multiplication dynamics suggests a counter mechanism of division- which is further suggested by a significant relation of merozoite numbers with schizont size at the onset of division.
c. Nutritional deprivation caused increase in nuclear volume and decrease in merozoite number. For the most part, the experiments that are presented in this manuscript support the conclusion of the authors. The data are presented in a concise and clear manner. However, some clarification and a couple of experiment (listed below) would improve this manuscript.

Major comments:

1. The authors generated at least 3 transgenic lines for this study, But the did not present any genetic validation of the lines in the manuscript. For completeness, I recommend to provide genetic validation (either pcr genotyping or whole genome sequencing) of the lines that were generated and used in this study in the supplement.

Our study exclusively used episomal expression of the respective fluorescent reporter (H2B-GFP, NLS-mCherry, and cytoplasmic GFP). As is customary in the field resistance to selection drugs and distinct fluorescent signals are assumed to sufficiently validate the presence of the plasmids. We now added the schematic maps of the plasmids in a new Fig. S1 to make our approach more visually clear.

2. In the H2B-GFP lines, the authors episomally GFP-tagged histone 2B to label the nuclear chromatin for both P. falciparum and P. knowlesi. This provides a very useful parasite line which enables the live time-lapse microscopy. Using these parasite lines, the authors first show that despite having a 24 h IDC in P. knowlesi vs 48 h in P. falciparum, both these parasites have a similar duration of the schizont stage (8.s vs 9.4 h). My concern here is whether this GFP-tagging is influencing the growth dynamics as in slowing down the P. knowlesi parasites. However, if that was the case authors should have seen that for P. falciparum too. Also, for the P. falciparum parasites that episomally express cytosolic GFP and Nuclear mCherry have a higher number of merozoites compared to the H2B-GFP P. falciparum and the authors speculate this is probably because of not tagging Histone 2B. Given this, it is important to show that none of the H2B-GFP parasites show any significant fitness cost due to GFP tagging of histone. I recommend a simple experiment to compare the multiplication rate of H2B-GFP lines to the parental lines in identical growth conditions. This suggested experiment was described in PMID: 35164549 to determine fitness cost of knockout lines. This experiment is vital for validation of the H2B-GFP lines and subsequent interpretation of the data that were presented in this manuscript.

We thank the reviewer for this excellent suggestion. To validate our lines further we now have carried out multiplication rate measurements similar to the one described in the designated publication for all the used lines alongside their parental strains (Fig. S2). We found no significant differences in between the wild type and the episomally expressing parasite lines (lines 131ff), which gives us confidence that episomal expression of tagged proteins do not significantly alter growth dynamics in these cases.

3. The authors used the microtubule live cell dye SPY555-Tubulin in P. falciparum to validate the findings presented in 1D and 1E. They did not do that for P. knowlesi. If there is no unsurmountable technical difficulty, I suggest doing the same with P. knowlesi. This will also address the concern that I have pointed out in #1.

Thank you for this suggestion. We have now generated the requested data with *P. knowlesi*, added it to what is now Supplemental Figure 3 and included it in our new analysis (Fig. 2I-J). The numerical values align well with the observations made when measuring schizont stage dynamics with the H2B-GFP expressing *P. knowlesi* line (line 158). A notable difference is that the Tubulin data strongly support the (refined) counter model, while the H2B data alone allow no distinction.

4. The data in Figure 3 shows that merozoite number does not depend on host cell diameter. My question here is, were these data collected using different donor blood? Or were this measured from different biological replicate? These are not clear from the writing. I am not sure about whether blood from various donor would have on the data, however, different preparation of the cells across various biological replicate

will have some effect on host cell diameter hence on data. State if these were collected from independent biological replicates and about the donor blood.

The data results where indeed collected from three independent biological replicates using different donor blood batches. This is now stated in the figure legend. The batches displayed no difference in RBC diameter.

5. It is interesting to see that nutrient-limited conditions increase average nuclear volume but less merozoite numbers. In this experiment, as I understand, complete media was diluted 0.5x, which basically diluted every component of the media by half. From this experiment I can see nutritional deprivation as a whole having an effect and supports the counter mechanism, it would be intriguing to see if there is any effect of a particular nutrient have any effect on progeny division. For example, parasites can be grown in amino acid deprived media (except isoleucine) which makes the parasites fully dependent on host cell amino acids. This sort of specific nutrient deprivation will probably allow the authors to probe for specific nutrients that plays role as counter mechanism factor.

This is indeed a very exciting direction we would like to investigate in more detail in follow-up studies. Our aim for this study was to confirm that nutrient deprivation actually affects “counting” and to provide a workflow to investigate individual nutrients. In the meantime the Mota group, in a study we now cite in the discussion (lines 507ff), actually reported that isoleucine (and possibly methionine) levels are linked to progeny number. A follow-up on this topic using our strains and methodology is certainly worthwhile but requires more detailed analysis in the future.

Minor comments:

1. P. knowlesi is sometimes just written as knowlesi. Please, write P. Knowlesi.

Has been corrected.

2. Supplemental figure 1D, missing x-axis label.

We added the x-axis label.

3. In line 105, define N/C.

Done.

4. In line 205, I assume the authors mean episomally, not episomally.

Thank you for pointing this out. We have replaced “ectopically” with “episomally” throughout the text.

5. In line 275, Duration of Schizont stage was slightly....

Has been corrected.

6. All 'ml' or 'µl' should be 'mL' or 'µL'.

Changes have been made.

7. Define iRPMI.

We added a definition (line 610).

8. In line 475, replace 'as' with 'and'.

Done.

Reviewer #3 (Significance (Required)):

The factors that regulate the number of progenies in malaria parasites remain unknown. While there are few previous studies attempting to answer the question, those studies were done on fixed stained cells. In this study, the authors used genetically modified fluorescent P. falciparum and P. knowlesi parasites that enable live microscopy. These parasites coupled with super-resolution time-lapse microscopy the authors attempt to investigate the mechanism(s) at play in regulating progeny division. This manuscript provides data to suggest that external resources might have some role in progeny division and supports the counter mechanism. More careful validation of the transgenic lines that were used to collect data presented needs to be more systematic and rigorous.
